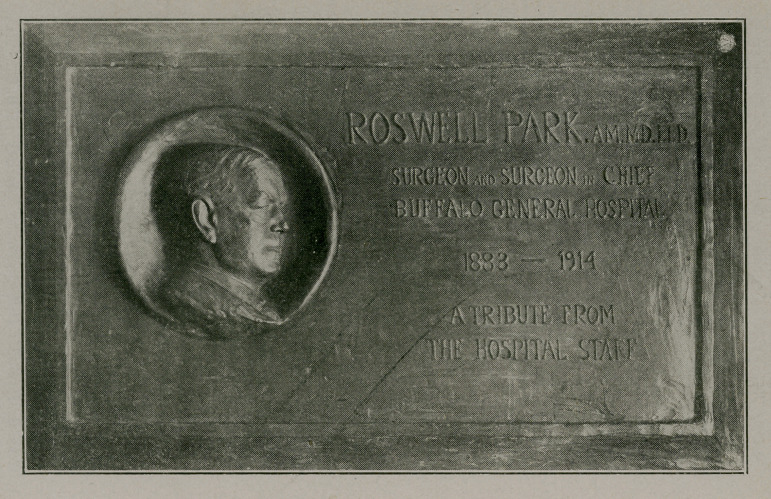# Memorial Tablet

**Published:** 1915-12

**Authors:** 


**Memorial Tablet** to the late Dr. Roswell Park in Buffalo General Hospital. (Illustration by courtesy of Buffalo Express.)

## Figures and Tables

**Figure f1:**